# No evidence for strong cytonuclear conflict over sex allocation in a simultaneously hermaphroditic flatworm

**DOI:** 10.1186/s12862-017-0952-9

**Published:** 2017-04-20

**Authors:** Nikolas Vellnow, Dita B. Vizoso, Gudrun Viktorin, Lukas Schärer

**Affiliations:** 1 University of Basel, Zoological Institute, Evolutionary Biology, Basel, Switzerland; 2 University of Innsbruck, Institute of Zoology, Innsbruck, Austria

**Keywords:** Genomic conflict, Cytonuclear conflict, Sex allocation, Cytoplasmic male sterility, Animal, Simultaneous hermaphrodite

## Abstract

**Background:**

Cytoplasmic sex allocation distorters, which arise from cytonuclear conflict over the optimal investment into male versus female reproductive function, are some of the best-researched examples for genomic conflict. Among hermaphrodites, many such distorters have been found in plants, while, to our knowledge, none have been clearly documented in animals.

**Methods:**

Here we provide a quantitative test for cytonuclear conflict over sex allocation in the simultaneously hermaphroditic flatworm *Macrostomum lignano*. We used a quantitative genetic breeding design, employing pair-wise crosses of 2 × 15 independent inbred lines, to partition the phenotypic variance in several traits (including sex allocation) into its nuclear and cytoplasmic components.

**Results:**

Although the nuclear genetic background had a significant effect on all traits analyzed, we found significant cytoplasmic genetic variation only for ovary size, there explaining just 4.1% of the variance. A subsequent statistical power analysis showed that the experimental design had considerable power to detect cytonuclear interactions.

**Conclusion:**

We conclude that there were no strong effects of cytonuclear conflict in the studied populations, possibly because the usually compact mitochondrial genomes in animals have a lower evolvability than the large mitochondrial genomes in plants or because the sampled populations currently do not harbor variation at putative distorter and/or the restorer loci.

**Electronic supplementary material:**

The online version of this article (doi:10.1186/s12862-017-0952-9) contains supplementary material, which is available to authorized users.

## Background

It has been recognized since the late 1960s that many evolutionary phenomena cannot be understood except in the light of genes being selected to selfishly increase their own representation in a population, also called the gene-centered view of evolution [1–3]. In fact, over the course of the last decades it has become evident that even genes within the same individual can be in conflict with each other [4–6]. Empirical examples for such genomic conflict—sometimes called ‘intragenomic conflict’, although that term poorly reflects that genes within one individual may reside on different genomes—are now manifold and range from driving B-chromosomes [7], over transposable elements [8] and driving sex chromosomes [9], to cytoplasmic sex allocation distorters [4, 10, 11].

Cytoplasmic sex allocation distorters emerge from cytonuclear conflict over optimal investment into male versus female reproductive function [4, 12, 13]. They are a common phenomenon, since they result from the almost ubiquitous maternal inheritance of cytoplasmic genetic factors, including mitochondria and chloroplast genomes, cytoplasmic endosymbionts, and vertically-transmitted parasites [14]. Because of their (near) exclusive transmission through female gametes [15], any newly emerging cytoplasmic factor that increases the fitness via maternally derived offspring will increase in frequency in the population, even if it at the same time harms male reproduction [4, 12]. In fact, in cases where the fitness via maternally derived offspring trades off with the fitness via paternally derived offspring, any cytoplasmic mutation that will reduce investment into the male function will spread, because it simultaneously increases investment into the female function and therefore its own fitness. For nuclear genes, however, equal investment into male and female function often is the evolutionary stable strategy—at least under the commonly made assumptions of random mating and large population size [16]—resulting in cytonuclear conflict over sex allocation.

As a result of cytonuclear conflict, cytoplasmic sex allocation distorters are thought to emerge and spread, only to be counteracted by nuclear restorer alleles that restore sex allocation towards the nuclear optimum. According to Hurst et al. [13] cytonuclear conflicts (and genomic conflicts in general) may bring about different outcomes. The conflict can disappear when a suitable restorer allele goes to fixation and the distorter allele is lost as a result, either via drift or via pleiotropic costs. Subsequently, even the restorer allele can be lost again if it bears fitness costs itself, so that no signs of the conflict will remain visible in the population and it will be hard to tell that the conflict had ever manifested itself. Another possible outcome is a stalemate, in which the distorter does not manage to “win”, but neither is the arisen restorer powerful enough to push the sex allocation all the way back to the nuclear optimum and hence to drive the distorter to extinction. Instead the population will be polymorphic for the distorter and restorer loci [11]. Moreover, cytonuclear conflict can also lead to the extinction of the whole population if a distorter spreads too fast for a restorer allele to emerge in time before extinction and the absence of male function comprises population fertility. This outcome seems to be most likely for those selfish elements that also have a strong negative effect on population growth rate, as is the case for y-linked meiotic drive genes [1], but it can also occur in populations of self-incompatible hermaphrodites in which a cytoplasmic sex allocation distorter rapidly spreads to fixation [17].

Empirically, it will be difficult to find evidence of conflict unless the studied population is in a transitional state or finds itself in a stalemate situation, with polymorphisms at either the distorter or the restorer loci (or possibly both). Since changes in sex allocation caused by cytoplasmic sex allocation distorters can have strong fitness effects, both distorters and restorers may sweep quickly to fixation and no traces of conflict will be found, despite the cytonuclear conflict being latently ever-present in species with uniparental inheritance of cytoplasmic genes.

Cytonuclear conflict over sex allocation manifests itself in different forms in gonochorists (separate-sexed organisms) and hermaphrodites. While in gonochorists the bone of contention is the investment into male versus female offspring (i.e. the sex ratio), it is the resource investment into an individual’s male versus female function that is at stake in hermaphrodites.

In gonochoristic animals most known examples of cytonuclear conflict over sex allocation are caused by cytoplasmic genetic elements other than mitochondria [10, 13]. For instance the intracellular alphaproteobacterium *Wolbachia*, which is common in many arthropod species, is transmitted vertically through the cytoplasm of the egg and thus also has uniparental inheritance. It can cause diverse phenotypes in its hosts, such as feminization of offspring, induction of parthenogenesis or killing of sons for the benefit of the daughters, in order to improve its own transmission at the cost of host fitness [18].

To our knowledge there is only one clear example of complete male sterility induced by mitochondria in animals, namely in *Drosophila melanogaster*, in which crosses with the cytoplasm from one specific population, but nuclear genomes from different populations, are male-sterile due to an impaired sperm differentiation [19, 20]. However, it is currently unclear whether this mutation—a single amino acid change in the cytochrome b protein—is just plain deleterious, or whether it conveys a transmission advantage to the mutant mitochondria. Furthermore, Patel et al. [21] found a mutation in subunit II of the mitochondrial cytochrome c oxidase gene that reduces male fertility in *D. melanogaster*, presumably because it impairs sperm development and motility, with no detectable effect on female fecundity. This reduced male fertility could be restored by several different nuclear genetic backgrounds, consistent with a ‘sex-specific selective sieve’ [22]. It seems unlikely, however, that this is the result of cytonuclear conflict over sex allocation, since there was no detectable transmission advantage to the mutant mitochondria [21].

Among simultaneous hermaphrodites, most of the evidence for cytonuclear conflict has been discovered in plants. Here it is usually a mitochondrial sex allocation distorter that leads to a male sterile phenotype, which is often evidenced as a failure to develop and/or mature the male reproductive tissues. This phenomenon is also called cytoplasmic male sterility (CMS) and male sterility has been found to be associated with an increase of ovule production in male sterile plants, presumably because of trade-offs between male and female function [23–25]. CMS is a common occurrence in angiosperms with 10% of the species exhibiting high frequencies of male sterile individuals due to one or several mitochondrial male-sterility genes [26]. Furthermore, as a result of CMS the sexual system of plants can change from hermaphroditism to gynodioecy, i.e. with the simultaneous presence of hermaphrodites and females (essentially male-sterile hermaphrodites), but no males in the population [27]. Gynodioecy has been described in 350 species from 39 plant families [10].

CMS can manifest itself in widely different morphologies, such as the complete absence of male reproductive organs, meiotic failure, or abortion of pollen at diverse developmental stages. And while many studies find very strong effects of mitochondrial sex allocation distorters, leading to complete male sterility [28, 29], these effects can also be of a more quantitative nature, in which the allocation into the male function is only partially reduced [11]. In fact, every sex allocation distorter is expected to spread, no matter how strong its effect on sex allocation, as long as it results in increased seed production, so that one can expect many small (and thus possibly difficult to observe) negative effects on male allocation in natural populations [10].

Given how many times CMS has been observed among hermaphroditic plants, it is surprising that mitochondrial CMS has never been documented in hermaphroditic animals, particularly since the same fundamental cytonuclear conflict over sex allocation should also be present in these organisms. In fact, gynodioecy, which is thought to be the result of CMS in plants (see above), is very rare in animals. In his careful review on sexual systems among animals Weeks [30] mentions only nine gynodioecious animals, despite a comprehensive literature research. Most of these species are not well studied, so even their classification as gynodioecious should be viewed with some caution, as it can be difficult to distinguish gynodioecy from sex change or from simultaneous hermaphroditism with pure-sex life-stage. One interesting and plausible example of CMS occurs in the Caribbean reef coral, *Porites astreoides*, which has been described as gynodioecious [31]. It is, however, not known whether mitochondria or other cytoplasmic endosymbionts are involved in this case, because in *P. astreoides* not only mitochondria, but also ectoderm-associated bacteria [32], endosymbiotic dinoflagellates [33] and possibly also apicomplexans [34] are transmitted vertically from mother to offspring. It could arguably be in the interest of all of these symbionts to decrease their hosts’ male allocation in favor of an increased female allocation. So on balance, there is a striking paucity of mitochondrial CMS as well as gynodioecy in general in the literature about hermaphroditic animals.

Several explanations for the lack of mitochondrial CMS in hermaphroditic animals have been proposed [35], of which the two main ones are that (i) either hermaphroditic animals have not received enough scientific attention in this regard or that (ii) the mitochondrial genome is less likely to generate sex allocation distorters in animals compared to plants, possibly because of its smaller size, lack of recombination, and lack of gene rearrangements [13, 24]. With respect to the first point, sexual morphology is indeed readily observable in the flowers of angiosperms, as these organs are very extrovert and evident, while in hermaphroditic animals ovaries and testes are often hidden inside the body, and thus more difficult to observe. Therefore male-sterile phenotypes are probably easier to spot in angiosperms (by lack of stamen and pollen) than in most hermaphroditic animals, although some transparent hermaphrodites, such as ctenophores, chaetognaths and many flatworms should also allow for easy identification of male-sterile phenotypes (e.g. lack of testes and/or male copulatory organs). Also, many angiosperms are of commercial interest and plant breeders commonly cross different varieties or even species in order to produce male-sterile individuals with increased seed production, which makes the detection of sex allocation distorters more likely and lucrative [36]. However, many male-sterile phenotypes in plants have been found in natural populations as well, a phenomenon of which already Darwin was aware [23].

Concerning the second point, animal mitochondrial genomes are indeed small compared to those of plants. They commonly only contain 37 encoded genes (range: 14–53) and have a rather narrow size range between 11 and 32 kb [37]. In contrast, the size of plant mitochondrial genomes ranges from 200 to 11,000 kb and large parts of these genomes have been shown to consist of non-coding regions [38]. And although non-coding regions also exist in animals, we still have a fairly limited understanding of the function of these regions [37]. Furthermore, the mitochondrial genome in plants experiences more gene rearrangements [39] and recombines more often [40]. It therefore appears possible that a greater evolutionary potential of plant mitochondria might make the emergence of new sex allocation distorter mutations more likely in plants compared to animals.

### Objective

Here we test for the presence of cytoplasmic sex allocation distorters in a simultaneously hermaphroditic animal, by performing a quantitative genetic breeding study in the free-living flatworm *Macrostomum lignano*. Specifically, we partitioned the phenotypic variance in several morphological traits (including sex allocation) into its nuclear and cytoplasmic components. The experiment was conducted under the rationale that, even if there are no drastic signs of CMS, such as complete male sterility, there might still be quantitative effects of cytoplasmic genetic elements on sex allocation, as different cytoplasmic distorters and nuclear restorers interact to determine the resulting sex allocation.

## Methods

### Study organism

The free-living flatworm, *Macrostomum lignano* Ladurner, Schärer, Salvenmoser, Rieger 2005 (Macrostomorpha, Rhadbitophora, Platyhelminthes) is a member of the intertidal meiofauna of the Adriatic Sea and the Eastern Mediterranean basin [41]. These small (adult length ~ 1.5 mm) worms can be cultured in the laboratory (at 20 °C, 14:10 h light:dark, and 60% humidity) in glass Petri dishes, in either artificial seawater (ASW) or the nutrient-enriched f/2 algal culture medium [42], with the diatom algae *Nitzschia curvilineata* as the sole food source [41, 43]. *M. lignano* is an obligatory outcrossing simultaneous hermaphrodite with an ~18d generation time [44] and shows frequent reciprocal mating [45]. Its highly transparent body permits detailed measurements of internal reproductive structures (see below), and thus allows us to obtain non-invasive estimates of sex allocation [44, 46].

### Establishment and maintenance of the inbred DV lines

We have previously given brief accounts of the establishment of certain inbred lines used here, namely DV1 [46]—the line used for the *M. lignano* genome project [47]—and DV3, DV8, DV13, DV28, DV69, and DV71 [48]. Here we provide a fuller account of the establishment of these and many additional lines.

We initiated the inbred lines in January 2004 by assembling 240 pairs of virgin worms from a range of outcrossed laboratory cultures started from different source populations (see Table 1), each pair from the same source population. The straight-line distances between these source populations range from ~0.2 km (PS to X), over ~2.5 km (PS to P1), to ~10 km (PS to UV). Although a larger geographic sampling range might have been preferable to maximize the diversity of cytoplasmic vs. nuclear genetic backgrounds, this range covered the entire confirmed distribution of *M. lignano* at that time [41]. From each founding pair, a line was initiated from the maternal offspring of only one member, which—assuming maternal transmission and no heteroplasmy (see the “Sequencing of mitochondrial *nad2* and *cox1-cytb* fragments” section in the Results)—associates one specific cytoplasmic genotype (cytotype) with one subsequently inbred nuclear background. In order to reduce the probability of a line dying out, we maintained up to six copies of each line.

**Table 1:** The number of initiated inbred DV lines, those used in the experiment, and those surviving until the current moment, grouped by their source populations in the Northern Adriatic Sea [41]

Source (Site)		Initiated (n) (in 2004)		Used DV lines (in 2006)				Surviving DV lines (currently)			
Lignano Sabbiadoro 1995 (P1)		30		22^f^, 39^g^, 57^g^				18^g^, 22^f^, 27^g^, 39^g^, 41^g^, 57^g^, 71^e^, 83^g^			
Lignano Sabbiadoro 2002 (P1)		7		na				25^d^			
Isola di Martignano 2002 (X)		8		47^i^				47^i^			
Isola di Martignano 2003 (PS)		90		26^c^, 28^c^, 33^c^, 49^j^, 67^a^, 68^b^, 72, 81^c^, 84^j^				16^c^, 26^c^, 28^c^, 33^c^, 49^j^, 51^c^, 65^c^, 67^a^, 68^b^, 81^c^, 84^j^			
Bibione 2003 (UV)		105		1^c^, 3^d^, 6^d^, 8^d^, 12^d^, 13^c^, 14, 29^c^, 35^d^, 40, 44^d^, 46^d^, 50^d^, 61^d^, 69^d^, 75^h^, 76				1^c^, 3^d^, 6^d^, 8^d^, 10^c^, 12^d^, 13^c^, 20^g^, 29^c^, 31^c^, 35^d^, 37^c^, 44^d^, 46^d^, 50^d^, 61^d^, 69^d^, 75^h^			
Total (n)		240		30				39			
The letters indicate the different mitochondrial haplotypes of DV lines, with the following polymorphic bases and resulting amino acid substitutions (base positions are given with respect to the start of the sequences for each fragment):											
Haplotype	350	373	408	421	587	40	127	148	386	441	528
a	A	C	C	A	G	A	A	C	T	C	T
b	A	C	C	T	G	A	A	C	C	C	T
c	A	C	C	T	G	A	A	C	T	C	T
d	A	C	T	T	G	A	A	C	T	C	T
e	A	T	C	A	A	A	A	C	T	C	T
f	A	T	C	A	G	G	A	C	T	A	C
g	A	T	C	A	G	G	A	C	T	C	C
h	A	T	C	A	G	G	A	C	T	C	T
i	A	T	C	T	G	A	A	T	T	C	T
j	G	C	C	T	G	A	G	C	T	C	T
Encoded amino acids	I/V	F	A/V	L/F	V/I	L	W	R	F/S	F/L	V
Open reading frame	*nad2*	*nad2*	*nad2*	*nad2*	*nad2*	*cox1*	*cox1*	*cox1*	*cytb*	*cytb*	*cytb*

Underlined DV lines were used in the experiment but are no longer available. Note that DV stands for Dita Vizoso, who was mainly responsible for their establishment. The table footer indicates the observed haplotypes in the sequenced genes (see also main text)

During the first 15 generations of inbreeding each copy was started with only two juvenile offspring per parental cross, so that the resulting parents of the next generation were necessarily full-sibs and the resulting offspring therefore stemmed from maximal biparental inbreeding. Moreover, in order to maximize the loss of genetic diversity, all copies of each line were made from a single parental pair whenever possible. Despite this replicated breeding scheme we lost a substantial number of lines over several generations. From generation 15 onwards we therefore started a second breeding scheme with each copy within a line being started with three, rather than just two, juvenile offspring from the same parental group. So while the resulting parents of the next generation could have either been full- or half-sibs, the resulting offspring still stemmed from substantial biparental inbreeding. The experiment reported here was initiated in generation 20 by taking worms from this second inbreeding scheme (see next section). As a result of these two breeding schemes we can expect inbreeding coefficients of F_t_ > 0.97 [49], representing a very high level of inbreeding.

For completeness, we also briefly report how these lines have since been kept, and which lines are still being maintained to date (Table 1). The second inbreeding scheme was kept until generation 24, when we switched to maintaining two copies (A and B) from then onwards, each generation being initiated with up to ten offspring from each parental group, thus maintaining a small effective population size and a high level of inbreeding. Again, whenever possible, both copies descend from the same parental group. Moreover, at irregular intervals we further pass these lines through a population bottleneck by starting the populations with only up to five individuals. Specific inbred lines are then expanded whenever they are needed for experiments, with the stock cultures always maintained at these small population sizes. The inbred lines (and also the experimental crosses) were cultured in 6- or 24-well tissue culture plates (TPP, Switzerland), with parental pairs and triplets held in the latter and larger parental groups in the former.

### Crossing design

**Fig. 1:**
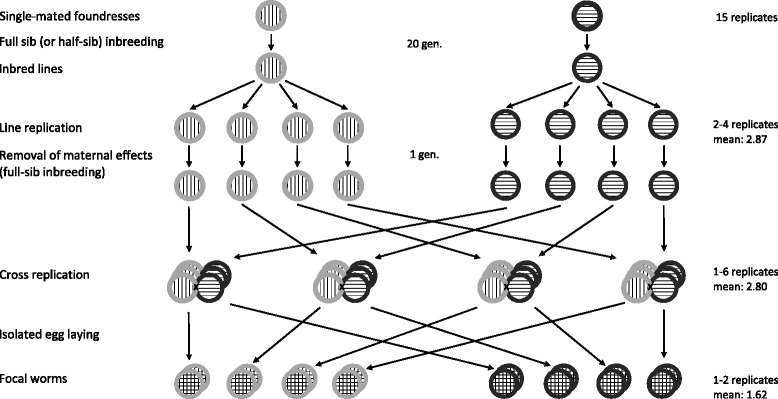
Crossing scheme used in this study (shown here for one of the 15 independent pairs of inbred line crosses) in which the *stripe pattern* of the inner circular area represents the nuclear genome and the shade of *gray* of the outer rim indicates the cytotype. Note how both lines were replicated and grown independently for one generation to account for maternal effects in the analysis and how the resulting offspring of the line crosses share the (approximately) identical nuclear genome, but differ in the cytotype depending on which line is the maternal parent.

The rationale of the experimental design takes advantage of the fact that (i) in a simultaneous hermaphrodite we can obtain maternal offspring from both parents (and thus potentially different cytotypes) of a specific cross and (ii) recombinant F1 offspring resulting from a cross between parents from two highly inbred lines inherit (almost) identical nuclear genetic material (as segregation and recombination cannot introduce new combinations among the gametes of these parents). Together this means that we can obtain individuals that may differ in the maternally inherited cytoplasmic genes, but are (nearly) identical in their nuclear genes. This permits to study if the maternal background affects any traits, as expected under cytonuclear conflict over reproductive allocation. One potential caveat of this approach, however, is that there could also be maternal effects linked to a common rearing environment if the parents from one line have grown up together (i.e. maternal effects could be confounded with cytotype effects). The design that we employed here takes all these points into consideration (Fig. 1).

In order to control for maternal effects, we used a split brood design to produce, initially, up to six independently grown pairs per DV line (up to four of which were actually used, see below). Specifically, we took, for each line, up to 12 offspring from a single parental group (containing two or three parents) and distributed them in up to six pairs in wells on 24-well plates. These pairs constitute our line replicates, having the same cytotype but potentially different maternal effects. We then collected up to six offspring per line replicate and allowed them to grow in isolation in separate plates (day 1). These worms were to become the parents in our crossing experiment. We randomly selected pairs of DV lines that had enough worms and enough line replicates to become our line crosses (note that only one line cross is depicted in Fig. 1). Within each line cross, we chose randomly two to four (of the initially up to six) line replicates to use in the further experiment, which were in turn replicated up to six times, in what we call the actual cross replicates (see Fig. 1). In order to distinguish the two parents within a pair, we dyed one parent by immersing it for 24 h in a 5.6 mg/mL solution of the food dye New Coccine in ASW (day 19), a dosage at which no adverse effects on the mating behavior are observed [50]. Which worm was dyed was decided using a restricted randomization approach. We produced and randomly distributed 277 pairs of 20 line crosses onto 24-well plates (day 20) and allowed the parents (one dyed and one undyed) to mate for three days, after which time we isolated them for egg laying (day 23) in order to be able to collect offspring separately from each maternal cytotype. These offspring were to become our focal worms (Fig. 1). We collected up to two such focal worms per laying parent (i.e. up to four per pair) and isolated them in 24-well plates (day 34).

In order to allow these focal worms to interact with a partner while keeping the resulting variation to a minimum, we used worms from what we call the tester DV line (DV20) from the same generation. We dyed said testers using the procedure described above, and added them to the focal worms (day 39). After five days (day 44), we re-dyed the testers (as the dye washes out eventually), and placed them with the focals in fresh plates (day 45). Finally, we measured the morphology of the focals, as described in the following subsection (days 50 and 51). In the end, we obtained enough focal worms (*n* = 391) for 15 of our initial 20 line crosses stemming from 241 crossings (i.e. actual parental pairs) of which in nine cases only one parent produced offspring.

### Morphological measurements

The morphological measurements were taken as previously described [44, 51]. Briefly, we anaesthetized worms with MgCl_2_ and squeezed them dorsoventrally between a glass slide and a hemocytometer cover glass, separated by a 35 μm plastic spacer. We then took digital micrographs at 40-400× with a digital video camera (DFW-X700, Sony Broadcast & Professional, Köln, Germany) attached to a Leitz Diaplan compound microscope (Leica Microsystems, Wetzlar, Germany). We used the image capture software BTV Pro (Bensoftware) to acquire the images and the pictures were analyzed with the public-domain image-analysis software Object-Image 2.09 (developed by Norbert Vischer, University of Amsterdam). Specifically, body size (the total body area), testis size (the sum of both testis areas), ovary size (the sum of both ovary areas), seminal vesicle size (the area of the seminal vesicle) and eye size (the mean of the area of both eyes) were measured this way. Sex allocation was calculated by dividing testis size by the sum of testis size and ovary size [51].

### Statistics

To test for effects of the cytotype as well as the nuclear genetic and maternal background on the different morphological measures a linear mixed model was fitted with the *lme4* package in R (version 3.2.4) using restricted maximum likelihood in order to extract unbiased variance estimates of the random effects [52, 53]. Note that because of the details of the used algorithms the estimate for a random effect variance will be zero in cases when the true random effect variance is very small or negative. In these cases the random effect can be considered as contributing no significant amount of variance [54]. Response variables were body size, testis size, ovary size, sex allocation, seminal vesicle size and eye size. All response variables were log- and z-transformed to meet the normality assumption for residuals and to facilitate model convergence, respectively. Only an intercept was used as fixed effect and random effects were fitted according to the nested structure of the experimental design, with cross replication nested within line replication, line replication nested within cytotype, and cytotype nested within line cross (Fig. 1 and Table 2). Random effects were removed in a stepwise fashion and the significance of the removed effect was tested by comparing the simpler model to the more complex model with a parametric bootstrap test with 20,000 iterations using the R package ‘pbkrtest’ [55]. Additionally, a plate ID random effect was included to account for variance among the well plates in which worms were held. The variance components to calculate the percentages of explained variance of the different factors were taken from the full model. The model assumptions of normally distributed and homoscedastic residuals were checked with normal q-q plots and residuals vs. fitted plots, respectively. For the eye size analysis 5 replicates had to be excluded because of missing eye size measurements. We chose not to correct for body size when analyzing testis, ovary, seminal vesicle and eye size as well as sex allocation since we assumed the absolute size of these organs to be more closely related to fitness than their body size corrected values (but see the Additional file 1 for an analysis that includes a body size correction, which is qualitatively very similar to the analysis presented in the main text). We included body and eye size as traits in the analysis to compare the results of sex allocation traits with and because mitochondria have been reported to be important components of the eyes of some flatworms including the Macrostomida [56].

**Table 2:** Shown are the percentages of explained variance by the different random effects (and their respective *p*-values) for the measured morphological traits

Factors	Body size	Testis size	Ovary size	Sex allocation	Seminal vesicle size	Eye size
Line cross	**28.0 (<0.001)**	**35.0 (<0.001)**	**24.4 (<0.001)**	**24.1 (<0.001)**	**28.8 (<0.001)**	**67.0 (<0.001)**
Cytotype	0.6 (0.17)	0.1 (0.66)	**4.1 (0.046)**	0.0 (1)	0.0 (0.68)	0.0 (0.83)
Line replication	4.4 (0.09)	0.0 (0.65)	0.0 (0.70)	0.6 (0.52)	1.4 (0.36)	**2.0 (0.019)**
Cross replication	2.3 (0.50)	0.2 (0.62)	0.0 (0.66)	0.0 (0.68)	0.0 (1)	4.9 (0.06)
Plate ID	0.8	1.6	1.1	3.0	5.2	0.0
Residual	63.9	63.2	70.4	72.3	64.6	26.1

The percentages of explained variance were calculated from the variance estimates in the full model and bold values indicate significant effects

In order to estimate the magnitude of the cytotype effect sizes that we could have detected with our experimental design, a post-hoc power analysis was performed. For this we i) simulated datasets for specific chosen values of the random effects (see below) using the same number of replicates for each factor level as in the original dataset, ii) tested for each of those simulated datasets the significance of the cytotype effect by comparing the model with cytotype nested within line cross with the reduced model only containing the line cross random effect by parametric bootstrapping (this time with 600 bootstrap iterations to work more time efficiently) and iii) calculated the proportion of tests that resulted in a significant *p*-value as an estimate of our statistical power. The chosen values for the variance components used for the simulations were assumed to be those from the model for ovary size (i.e. the values in the ovary column in Table 2), except that several different values for the cytotype effect size (i.e. the proportion of variance explained by the cytotype) were used (see the x-axis in Fig. 3) and consequently the residual variance changed as well. Here we chose to use the empirical values for ovary size because ovary size is a priori the most likely trait to be influenced by cytonuclear conflict, since it should translate most directly into an altered maternally derived fitness (note that when using the variance components from the model with testis size, very similar results were found). For each of the 11 different effect size values 1000 datasets were simulated, which adds up to a total of 11 × 1000 = 11,000 datasets. These simulations were also conducted in R version 3.2.4 [52].

In our discussion of effect sizes and statistical power we adhere to the conventions suggested by Cohen [57]. More specifically, we consider 0.01, 0.09 and 0.25 of the variance explained to represent small, medium and large effect sizes, respectively [57, 58] and we consider Hedge’s g values of 0.2, 0.5 and 0.8 to represent small, medium and large effect sizes, respectively [59].

### Mitochondrial genetic diversity of DV lines

In order to test whether worms were monomorphic within and showed mitochondrial genetic variation among the different DV lines, two mitochondrial DNA fragments, one within the *nad2* gene and one spanning the *cox1* and *cytb* genes, were amplified by PCR and sequenced. For each available DV line at least two individuals were sequenced. Moreover, in order to verify our assumption of maternal inheritance of the mitochondrial genome, two virgin individuals from inbred lines that differed in their *nad2* fragment were crossed and the parents as well as the offspring were sequenced. In some pairs one of the parents failed to produce maternal offspring, in which case we still analyzed the maternal offspring of the other parent. In total these added up to 40 instances of mitochondrial transmission from line crosses DV1xDV75 (six times two, and three times one maternal offspring per pair), DV39xDV69 (six times two, and six times one maternal offspring per pair) and DV71xDV84 (one time two, and five times one maternal offspring per pair).

Genotyping was performed as follows: For DNA isolation, individuals were immersed in 30 μl 100% Ethanol and kept at −20 °C for at least an hour. Ethanol was evaporated at 80 °C until dry, and 20 μl 10 mM Tris-HCL pH 8.0 containing 1 mg/ml Proteinase K (Roche, Mannheim, Germany) were added. Samples were incubated at 65 °C over night, followed by 15 min at 95 °C to deactivate Proteinase K. 0.5–1 μl of this isolate was used as template for a 20 μl PCR reaction (modified from *Caenorhabditis elegans* single worm DNA isolation method by H. Schulenburg, pers. comm.). PCR was performed using Q5 polymerase (New England Biolabs, Ipswich, MA, USA) using specific primers for the *nad2* fragment (MacNad2-F: TAAGATTAGTGGGAAAGATGGGAAG; MacNad2-R: AACAAACATAGAAAATGGGGGAATACC; fragment size: 380 bp) and the *cox1-cytb* fragment (MacCox1-F: GGTTTATCTGGTATGCCTCGTCG; MacCytb-R: CGCTCCTCAAAAAGACATCTG; fragment size: 680 bp). Cycling conditions were the same for both fragments: 30 s 98 °C, 35× (7 s 98 °C, 30 s 64 °C, 30 s 72 °C), 2 min 72 °C. Fragments were sequenced using the MacNad2-F and MacCytb-R primers, respectively.

## Results

### Effects of line cross, cytotype and other variance components

**Fig. 2:**
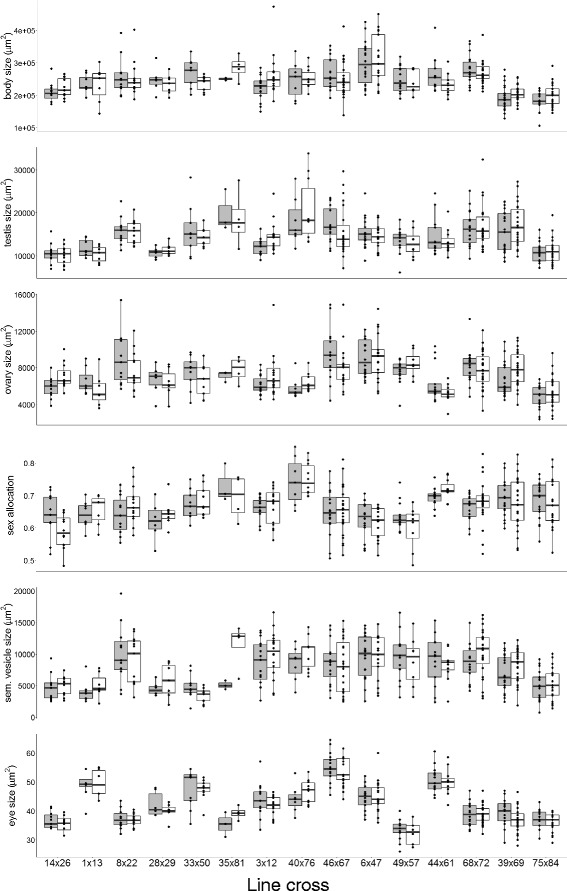
Effect of the specific line cross (e.g. DV14 x DV26) and their two respective cytotypes (box plots are *gray* for the first and *white* for the second line in each cross) on all the other measured traits. *Black dots* represent the individual measurements, the bottom and the top of the box represent the 25th and 75th percentiles, respectively, and whiskers extend to 1.5 times the interquatile range. Note that all traits are plotted on their original measurement scales for visualization (see also the “Statistics” section in the Methods).

Our analyses revealed significant line cross effects for all measured traits, as evidenced by the stepwise removal of lower level random effects and comparison of reduced to unreduced models (Fig. 2 and Table 2). This suggested that there was considerable genetic variation for all these measured traits, including sex allocation, among the nuclear genomes within/among the founding populations (Table 1), explaining between 24.1 and 67.0% of the observed phenotypic variance (Table 2). In contrast, the effect of the cytotype was not significant for most of the traits, except for ovary size (parametric bootstrap test, *p* = 0.046), where it explained only 4.1% of the variance (cf. ovary panel in Fig. 2). There was no apparent difference in the magnitude between the cytotype effects of traits measuring reproductive morphology, over which cytonuclear conflict seems more likely (i.e. testis size, ovary size, sex allocation and seminal vesicle size), and those measuring other morphological traits (i.e. body size and eye size, Fig. 2). Neither line replication, nor cross replication contributed a significant amount of variance for any of the traits, except for eye size, where line replication contributed 2.0% variance (parametric bootstrap test, *p* = 0.019), suggesting that there are no strong maternal effects for these traits.

### Post hoc power analysis

**Fig. 3:**
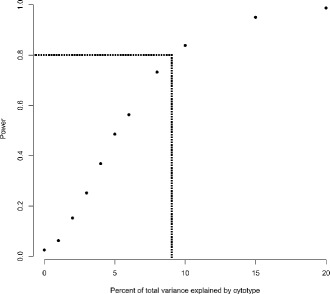
Power analysis for different simulated cytotype effect sizes (x-axis) assuming the same variance components as those empirically found for ovary size (see main text for rationale). Each *black dot* represents the proportion of 1000 simulations that resulted in a significant cytotype effect (with a significance threshold of 0.05). To achieve a power of 0.8 one needs a cytotype effect size of about 9% or more (*dotted line*).

Given the number of replicates we used for each factor level combination in this study and given the variance components that we have empirically found for ovary size, the cytotype effects would have needed to explain about 9% of the variance to be detectable with a power of 0.8 (Fig. 3). This is considered the desired power by convention in the statistical literature [57, 58] and we should thus have been able to reliably detect medium to large effect sizes of cytonuclear conflict with our experimental design.

### Sequencing of mitochondrial *NAD2* and *COX1*-*CYTB* fragments

Five and six polymorphisms were detected within the sequenced *nad2* and *cox1-cytb* fragments, respectively. Taken together, these 11 polymorphisms can be used to subdivide the 39 currently maintained DV lines into at least 10 groups with regard to their mitochondrial haplotype (see footer of Table 1). Six of the polymorphisms cause amino acid substitutions, of which four lie within the predicted *nad2* coding region, and two within the predicted *cytb* coding region (Table 1). Moreover, individuals belonging to the same DV line always carried the same *nad2* and *cox1-cytb* sequence, suggesting no mitochondrial variation within lines.

Many DV lines that were paired for the main crossing experiment differed in their *nad2* and/or *cox1-cytb* sequence, so that eight (DV8xDV22, DV33xDV50, DV35xDV81, DV46xDV67, DV6xDV47, DV49xDV57, DV39xDV69 and DV75xDV84) out of the 15 crosses used in this study were done between lines that clearly differed in their mitochondrial haplotype (irrespective of whether considering base or amino acid sequence), at least with respect to these gene regions. From the seven remaining crosses, five did not differ in the considered gene regions (DV1xDV13, DV28xDV29, DV3xDV12, DV40xDV76, DV44xDV61) and in the other two (DV14xDV26 and DV68xDV72) only one line could be genotyped, because the other line had in the meantime been lost.

Finally, in all of the 40 instances in which both the mother and her maternal offspring were sequenced for the *nad2* gene, the maternal mitochondrial genotype was inherited. This suggests exclusive maternal inheritance (or at least a low frequency of paternal inheritance) of the mitochondrial genome in *M. lignano*, thus fulfilling an important assumption of the predicted cytonuclear conflict.

## Discussion

### Short summary of results

Our study confirmed that there was, for all traits measured, considerable and statistically significant nuclear genetic variation in the sampled populations from which the inbred lines used here were derived [60]. Conversely, no significant variation due to the cytotype could be found in either sex allocation or the other measured traits, except for a fairly small, but statistically significant effect on ovary size. This was the case in spite of the fact that the experiment was statistically powerful enough to detect effects of medium to large size (i.e. cytotype effects that explain 9% of the observed variance or more), while the observed ovary size effect did not reach that threshold. Therefore we did not find evidence for strong sex allocation distorters in our experiment, which were predicted under the above-mentioned scenarios of cytonuclear conflict over sex allocation. We do not interpret the detected cytotype effect on ovary size as strong evidence for cytonuclear conflict over sex allocation, because of three reasons. Firstly, the effect was fairly small, especially when compared to the line cross effects. Secondly, the relevant *p*-value was just below the significance threshold of 0.05 and since we performed many tests in this study we cannot exclude the possibility that this represents a type I error (and any approach to correct for multiple testing would surely have rendered this effect non-significant). And lastly, the cytotype effect on ovary size might in part have been due to variation in body size, because for some of the crosses where the cytotypes differed most strongly, body size actually differed in the same direction (e.g. DV14xDV26, DV39xDV69, DV44xDV61 and DV46xDV67; cf. body size and ovary size panel in Fig. 2). In fact, when controlling statistically for body size the cytotype effect on ovary size disappeared, because of the strong correlation between body and ovary size (see Additional file 1). However, we think the fact that we did not find strong evidence for cytonuclear effects on sex allocation is very interesting and important, bearing in mind that there are, to our knowledge, no published studies looking at this phenomenon in simultaneously hermaphroditic animals so far and that the publication of such “negative” results is crucial for scientific progress [61–63]. In the following we discuss different aspects of our results in some more depth.

### Statistical power

Assuming that the magnitude of cytoplasmic effects on sex allocation is comparable to that of other cytoplasmic genetic effects recently summarized in a meta-analysis [59] we had good statistical power to detect cytotype effects. In their study Dobler et al. [59] found the average effect size to be medium (Hedges’ g: 0.49; 95% CI: 0.30–0.79), although many studies they analyzed also reported small cytoplasmic genetic effects. It is therefore possible that some small effects went undetected in our study. However, medium to large effects, or even cases of complete male sterility, which are often reported in the CMS literature for plants [11, 28, 29], should clearly have been detectable in our study with a high power, especially since we measured sex allocation quantitatively and with a well-established method that has previously revealed theoretically predicted phenotypically plastic adjustments of sex allocation to changes in the social environment [44, 46]. Our study also had considerable statistical power compared to many other published studies, as most studies in the behavioral ecology and animal behavior literature have a lower power to detect small or medium sized effects [64], compared to the power observed here (Fig. 3).

### Measured traits

Sex allocation distorters should be selected to increase the investment into the female function, for example, by reducing the investment into male function when there is a trade-off between the two sex functions. In *M. lignano* accumulating evidence suggests that there is indeed a trade-off between ovary and testis size [65, 66]. These morphological measures of sex allocation seem to reflect allocation to male and female function well, and testis size has been shown to correlate with the cell proliferation activity in that organ [67] and with the amount of sperm produced [68]. Moreover, testis size also predicts male sperm transfer success [69] and paternity success [70], presumably because worms with bigger testes manage to transfer more sperm per mating [70]. Given all these reasons one could therefore have expected a cytotype effect on testis size, on ovary size, and/or on sex allocation, if there indeed was variation at a sex allocation distorter locus in the sampled populations.

### Establishment of inbred lines

Of the 240 initiated inbred lines almost 200 went extinct because the worms failed to reproduce sufficient numbers of offspring to maintain the lines (cf. Table 1). The most parsimonious explanation for this seems to be inbreeding depression, which is a common phenomenon in the establishment of inbred lines [71, 72], especially if outbreeding is the normal reproductive mode, as likely is the case in *M. lignano* [44]. However, complete or extremely strong CMS could in theory also cause a failure to reproduce, e.g. if all individuals within a line were male sterile, so that there were no functioning sperm available to fertilize the eggs. In such cases any sex allocation distorter alleles causing a complete shut-down or extremely strong reduction of male fertility might have been lost due to the extinction of those lines, thus potentially explaining why we did not find complete male sterility in the surviving lines. Given that we almost never see completely male-sterile individuals in our outbred cultures stemming from the same source population as the DV lines, this, however, seems a rather unlikely scenario (LS and NV, pers. obs.), particularly since only a few functioning sperm cells would likely have been necessary to fertilize enough offspring to produce the next generation under the non-competitive inbreeding system we employed here.

### Stage of the evolutionary arms race

The lack of evidence for cytonuclear conflict on sex allocation could, of course, have resulted from the fact that we happened to sample the population(s) at a stage of the evolutionary arms race when there was no variation at either putative cytoplasmic sex allocation distorter loci or nuclear restorer loci [13]. Or in other words, the population(s) studied could have been sampled at a time point when either a nuclear restorer or a partial cytoplasmic distorter had just swept to fixation. To test if this is true it would have been useful to cross more distant populations (or even incipient species) of *M. lignano* and to investigate whether the resulting offspring suffers from reduced male allocation caused by cytoplasmic distorters. A period of independent evolutionary history of multiple populations might have made it more likely that putative nuclear restorer alleles from one population fail to restore sex allocation back to the nuclear optimum in response to putative sex allocation distorters of another population. Generating such crosses has revealed many latent CMS genes in plants [24, 29, 36] and cytoplasmic genetic effects studied with interspecies crosses are typically larger than in intraspecies crosses [59].

The inbred lines used in our experiment were sampled from 3 sites (Table 1) at only a few kilometers distance from each other. We currently have no knowledge about whether and, if yes, how much gene flow happens between those sites and when their most recent common ancestor lived. Assuming these to be partially diverged sub-populations with somewhat independent histories of cytonuclear conflict, several of the crosses might have shown cytotype effects, since they were established between inbred lines from different sites (e.g. crosses DV14xDV26, DV8xDV22, DV28xDV29 and DV33xDV50; cf. Fig. 2). However, our results suggested this was not the case. Furthermore, eight crosses we tested differed in their mitochondrial haplotype for the *nad2* and/or *cox1-cytb* gene fragments as well as the encoded amino acid sequence, of which two were sampled from different sites (DV8xDV22 and DV33xDV50), of which DV8xDV22 might possibly have showed some degree of cytonuclear conflict (Fig. 2).

Alternatively, crossing *M. lignano* worms from these Italian populations in the Northern Adriatic Sea with *M. lignano* we have recently been able to sample from Greek populations from the Sithonia peninsula in the Northern Aegean Sea might potentially be more promising for detecting cytoplasmic sex allocation distorters, since these populations likely have had more time to diverge. This was unfortunately not possible when our experiment was conducted, because the worms from Greece were sampled for the first time in 2013 (i.e. several years after our experiment was performed), and the establishment of new inbred lines is a lengthy and highly laborious process. Any follow-up experiments should ideally be informed by a better understanding of the geographical distribution of *M. lignano* and the connectivity between different populations of this species. However, since we do not know much about the connectivity between populations and dispersal abilities of this species there was no reason a priori to expect that cytoplasmic sex allocation distorters are not detectable within the geographic scale of the Italian populations.

### Low evolvability of mitochondrial genome

Another possible explanation for the lack of strong cytotype effects is that cytonuclear conflict over sex allocation may not easily manifest itself in animals in general, due to the generally rather compact mitochondrial genome of most metazoans. After the acquisition of mitochondria into the cells of eukaryotes and the subsequent coevolution between the mitochondrial and nuclear genomes, many mitochondrial genes were transferred to the nuclear genome, so that today most metazoan mitochondrial genomes are small in size (~16 kb with a range between 11 and 32 kb) and usually contain only 13 protein-coding genes, 22 tRNA genes, two ribosomal RNA genes and only very few non-coding sequences [73, 37, 74]. In such a compact, “streamlined” and non-recombining mitochondrial genome new mutations are more likely to affect essential functions, while mitochondrial genomes in plants have the “space and opportunity to form new coding sequences among which sterility-inducing genes may emerge” [24]. However, although metazoan mitochondrial genomes are often viewed as being small, having invariant gene content, and being solely responsible for ATP production, evidence is emerging for their involvement in cell signaling and differentiation, fertilization and apoptosis [75]. And despite being more evolutionarily conserved than plant mitochondrial genomes, at least some animal mitochondrial genomes show striking deviations from typical architectures, with introns and linear or multicircle mitochondrial DNA, as well as variable gene content (mainly due to number of tRNAs). Breton et al. [75] make a good case for a stronger appreciation of the taxonomic variability in metazoan mitochondrial gene content and organization, and their evolutionary implications.

Nevertheless, except for lacking the atp8 gene and having a slightly modified genetic code [37, 75, 76], flatworm mitochondrial genomes studied to date are fairly representative of other Metazoa, and preliminary data suggest that the mitochondrial genome of *M. lignano* closely matches that of other flatworms (A. Waeschenbach and T. Littlewood, pers. comm.). But, animal mitochondrial genomes have been reported to evolve over evolutionary times as short as ten generations in a seed beetle [77] and cytoplasmic genetic effects in general do not seem to be stronger in plants than in animals [59]. Therefore it seems premature to flat-out refuse the possibility of the emergence of sex allocation distorters in animals, solely based on the argument of their compact mitochondrial genome.

### Implications for evolutionary transitions between sexual systems

Explaining the apparent lack of mitochondrial CMS in animals may not only shed light on the evolution of genomic conflict, but it may also have implications for our understanding of the evolution of sexual systems. In particular, CMS has been invoked as a “kick starter” of the evolutionary transition from monoecy via gynodioecy to dioecy in plants [78–80], while the rarer evolutionary transition from hermaphroditism to separate sexes in animals seems to happen more often via androdioecy rather than gynodioecy [30]. If, as our results seem to suggest, cytonuclear conflict in animals indeed leads to CMS less often and/or less easily than in plants, because sex allocation distorters may evolve less often in the compact metazoan mitochondrial genomes, then this could help to explain the different patterns for evolutionary transitions in sexual systems between animals and plants.

## Conclusions

In our study we did not find evidence for strong cytonuclear conflict over sex allocation in a simultaneously hermaphroditic animal. It would be tempting to use this evidence as confirmation for the hypothesis that the compact metazoan mitochondrial genome is less prone to evolve selfish sex allocation distorters compared to those of plants, resulting in different patterns of evolutionary transitions between sexual systems in animals and plants. This conclusion would be somewhat premature, however, given how little we currently know about the reproductive biology, sexual systems and mitochondrial genomes in many metazoan phyla. It has become apparent in recent years that mitochondrial genomes are not just “passive bystanders of adaptive evolution” [75] and the highly conserved stretches of DNA as which they were seen until a few decades ago. Thus further studies of mitochondrial genomes and their evolutionary effects seem clearly worthwhile.

For example, crosses between more diverged populations (or incipient species) of hermaphroditic animals could potentially reveal latent cytonuclear conflicts, as they have often done in plants. Furthermore, effects of mitochondrial genetic variation on reproductive phenotypes could be explored with gene silencing approaches, which are beginning to be used for editing the mitochondrial genome [81]. Moreover, elegant experimental evolution studies, similar to the approach used by Kazancıoğlu and Arnqvist [77], could be used in suitable model organisms to follow the spread of sex allocation distorters and restorers in real-time.

## Additional file

Additional file 1: Statistically controlling for body size. (DOCX 21 kb)
