# Supplementary material for: No evidence for strong cytonuclear conflict over sex allocation in a simultaneously hermaphroditic flatworm
Source: BMC Evol Biol. 2017 Apr 20;17:103. doi: 10.1186/s12862-017-0952-9 (PMC5397761; doi:10.1186/s12862-017-0952-9)
Supplement: Additional file 1: — Statistically controlling for body size. (DOCX 21 kb) [file 12862_2017_952_MOESM1_ESM.docx]

**SUPPORTING INFORMATION**

**Statistically controlling for body size**

Since ovary size and all other traits were correlated with body size we repeated the analysis using body size as a fixed effect in a linear mixed model while keeping the random effects structure as reported in the main text. Random effects were again removed in a stepwise fashion and the significance of the removed effect was tested by comparing the simpler model to the more complex model with a parametric bootstrap test with 20000 iterations using the R package ‘pbkrtest’ (Halekoh and Højsgaard 2014) but with body size as fixed effect remaining in the model. The results (Table S1) were very consistent with the analysis in the main text (Table 2). The only important difference was that the cytotype here explained even less of the variance in ovary size (excluding variance explained by the fixed effect of body size) and that this difference was thus no longer statistically significant.

Table S1. Shown are the percent of variance explained (and p-values in brackets) by different random effects while controlling for body size as fixed effect in the model. For body size the standardized slope β and the corresponding p-value are reported. There are no major changes compared to the original analysis (Table 2 in manuscript) except that here the cytotype explains almost no variance for ovary size either. Note that here the percent of variance explained by the random effects are calculated based on the variance that is not already explained by the fixed effect, i.e. the variance after controlling for body size is scaled to 100 percent.

| Factors | Testis | Ovary | SA | Seminal vesicle | Eye size |
| --- | --- | --- | --- | --- | --- |
| Body size  (fixed effect) | **β=0.42**  **(<0.001)** | **β=0.59**  **(<0.001)** | **β=-0.21**  **(0.001)** | **β=0.47**  **(<0.001)** | **β=0.13**  **(<0.001)** |
| Line cross | **31.8**  **(<0.001)** | **21.9**  **(<0.001)** | **26.0**  **(<0.001)** | **26.4**  **(<0.001)** | **66.6**  **(<0.001)** |
| Cytotype | 0.0  (1) | 0.6  (0.53) | 0.0  (1) | 0.0  (1) | 0.0  (0.28) |
| Line replication | 0.0  (1) | 0.0  (1) | 0.3  (0.59) | 2.3  (0.20) | **2.2**  **(0.02)** |
| Cross replication | 0.9  (0.54) | 0.0  (1) | 0.0  (1) | 0.0  (0.68) | 4.9  (0.06) |
| Plate | 1.3 | 1.1 | 3.2 | 5.3 | 0.0 |
| Residual | 66.0 | 76.4 | 70.6 | 66.0 | 26.3 |

**References**

Halekoh, U., and S. Højsgaard. 2014. A Kenward-Roger approximation and parametric bootstrap methods for tests in linear mixed models – the R package pbkrtest. J. Stat. Softw. 59.
